# Comprehensive Two-Dimensional Gas Chromatography with a TOF MS Detector—An Effective Tool to Trace the Signature of Grape Varieties

**DOI:** 10.3390/molecules29091989

**Published:** 2024-04-26

**Authors:** Daniela Fonseca, Nuno Martins, Raquel Garcia, Maria João Cabrita

**Affiliations:** 1 Mediterranean Institute for Agriculture, Environment and Development & Institute of Research and Advanced Training, University of Évora, Pólo da Mitra, Ap. 94, 7006-554 Évora, Portugal; daniela.fonseca@uevora.pt; 2 Mediterranean Institute for Agriculture, Environment and Development & Global Change and Sustainability Institute, University of Évora, Pólo da Mitra, Ap. 94, 7006-554 Évora, Portugal; nmartins@uevora.pt (N.M.); raquelg@uevora.pt (R.G.); 3 Department of Crop Science, School of Science and Technology, University of Évora, Pólo da Mitra, Ap. 94, 7006-554 Évora, Portugal

**Keywords:** HS-SPME-GC × GC-TOFMS, flow modulator, varietal aroma composition, grapes, LDA

## Abstract

Varietal volatile compounds are characteristic of each variety of grapes and come from the skins of the grapes. This work focuses on the development of a methodology for the analysis of free compounds in grapes from Trincadeira, Cabernet Sauvignon, Syrah, Castelão and Tinta Barroca from the 2021 and 2022 harvests, using HS-SPME-GC × GC-TOFMS. To achieve this purpose, a previous optimization step of sample preparation was implemented, with the optimized conditions being 4 g of grapes, 2 g of NaCl, and 2 mL of H_2_O. The extraction conditions were also optimized, and it was observed that performing the extraction for 40 min at 60 °C was the best for identifying more varietal compounds. The fiber used was a triple fiber of carboxen/divinylbenzene/polydimethylsiloxane (CAR/DVB/PDMS). In addition to the sample preparation, the analytical conditions were also optimized, enabling the adequate separation of analytes. Using the optimized methodology, it was possible to identify fifty-two free volatile compounds, including seventeen monoterpenes, twenty-eight sesquiterpenes, and seven C_13_-norisoprenoids. It was observed that in 2021, more free varietal volatile compounds were identifiable compared to 2022. According to the results obtained through a linear discriminant analysis (LDA), the differences in volatile varietal signature are observed both among different grape varieties and across different years.

## 1. Introduction

Aroma is a very important organoleptic characteristic in food. Aromas are formed by a huge diversity of volatile compounds, such as esters, terpene compounds, C_13_-norisoprenoids, C_6_ compounds, benzene compounds, alcohols, and aldehydes, among others [1]. The volatile varietal composition is characteristic of the variety of grapes and comes mainly from the skin, but also from the pulp of the grapes [2]. Terpene compounds and C_13_-norisoprenoids are the main compounds responsible for varietal aromas and have been extensively studied in grapes in recent years [3]. Exploring varietal volatile compounds contributes to differentiating viniferous varieties. It allows for tracing the characteristics of each grape variety, identifying the origin of the grapes, and evaluating each variety’s potential for wine production [4,5,6].

Monoterpenes are varietal compounds (C_10_) responsible for fruity (citrus) and floral aromas, formed from two isoprene units [7]. According to Cabrita et al. 2007 [8], monoterpenes can be grouped into classes with structures like nerol, linalool, and geraniol. Thus, compounds such as α-terpineol and p-menthen-7,8-diol belong to the nerol class, while the linalool family includes compounds like linalool, pyran oxides, and 2,6-dimethyl-2,7-octadien-1,6-diol, and finally, the geraniol class comprises citronellol and 3,7-dimethyl-1,7-octadiol. Sesquiterpenes are (C_15_) compounds consisting of three isoprene units in which an isopentenyl pyrophosphate (IPP) molecule reacts with a geranyl pyrophosphate (GPP) molecule to form farnesyl pyrophosphate (FPP) [9]. According to Petronilho et al. 2014 [10] and Cincotta et al. 2015 [11], sesquiterpenes have antioxidant, antibacterial, and anti-inflammatory properties. The content of these compounds affects the organoleptic characteristics of the grapes; hence, in the case of wines, for example, α-bisabol and α-calacorene are responsible for wood aromas and α-copaene is responsible for a spice aroma [2,12]. C_13_-Norisoprenoids are a group of aromatic compounds derived from enzymatic oxidative cleavage performed using carotenoid cleavage dioxygenases [13,14]. α-ionone and β-ionone are C_13_-norisoprenoids widely found in grapes, and result from the oxidative cleavage of carotenes, namely α-carotene and β-carotene, respectively [15]. The oxidative cleavage of neoxanthine leads to the formation of a “grasshopper” ketone, which then undergoes enzymatic transformation and is finally catalyzed with acid to form β-damascenone [16,17]. β-damascenone is known for its fruity–floral notes or baked apple aroma [18].

These compounds are present in grapes in trace amounts (μg kg^−1^) and for their analysis a previous sample preparation step that enables the isolation and/or preconcentration of the analytes is mandatory [19]. Solid phase extraction (SPE) is the most used sample preparation technique for grape analysis. SPE was developed at the end of the 1970s to replace liquid–liquid extraction (LLE), since it requires high amounts of solvents and is time-consuming. SPE enables the concentration and purification of analytes through the use of a cartridge containing a selective sorbent [20]. Compared to the LLE technique, SPE is faster, has greater reproducibility, requires the use of a smaller amount of organic solvents, allows the extraction of several analytes simultaneously, and enables the isolation of compounds in different fractions [21]. Recently, the demand for more sustainable, solvent-free, and straightforward techniques has increased, leading to the development of solid phase microextraction, SPME [22]. SPME can be performed in two modes: headspace (HS) or direct immersion (DI). HS-SPME is the most frequently used, as it preserves the lifetime of the fiber [23]. This technique, developed in the 1990s, is highly advantageous because it does not require the use of organic solvents, and it is a simple and fast technique; so, it is promising for the analysis of varietal volatile compounds [24].

Due to the complexity of the volatile composition of grapes and the trace levels of those compounds, it is crucial to make use of advanced analytical techniques that enable the separation and detection of the compounds from the whole matrix (grapes). Indeed, gas chromatography is the most frequently used analytical technique for this purpose. However, varietal volatile compounds from grapes are structurally very similar; thus, the occurrence of the co-elution phenomenon is commonly observed, hampering unequivocal detection by means of only unidimensional GC [25]. Thus, comprehensive two-dimensional gas chromatography (GC×GC) has emerged as a powerful analytical technique for the analysis of complex samples and as a potential alternative to one-dimensional separation [26]. This technique is based on the use of two columns coated with different stationary phases and connected through an interface called a modulator [27]. According to Robinson et al.’s 2011 study [28], the GC × GC provides more confidence in qualitative and quantitative analyses, a high separation efficiency, a good ability to detect minor analytes, and information about the sample and its composition.

In fact, modulation is one of the crucial steps in GC × GC and can be performed using cryogenic modulators or flow modulators. During the modulation period, the modulator is responsible for collecting the fraction that comes out of the first column, focusing it, and injecting the fractions into the second column [29,30].

The modulation range of flow modulators is significantly larger compared to that of thermal modulators; for example, eluates containing compounds with vapor pressures corresponding to C_1_ to C_40_ alkanes can be modulated [31]. Other advantages of using flow modulators compared to cryogenic modulators are better repeatability, no restrictions on sample volatility, and lower maintenance costs; moreover, the use of flow modulators does not require cryogen liquid to carry out the modulation [32,33].

Thus, the GC × GC system requires the coupling of detectors that can provide high detection efficiency, low noise, and quick response with small sample volumes over a broad dynamic range [25]. Recently, Mass Spectrometry (MS) has emerged as a powerful technique, providing information about the chemical identity of molecules in complex matrices. The combination of HS-SPME with GC × GC-TOFMS techniques allows for the analysis of complex samples, even if the compounds of interest are present in trace amounts, as is the case of varietal volatile compounds in grapes [34,35].

To the best of our knowledge, the use of flow modulator-based GC × GC-TOFMS for the determination of varietal volatile compounds in grapes has never been explored. This innovative study intends to achieve a deep knowledge of the volatile profile of Trincadeira, Cabernet Sauvignon, Syrah, Castelão, and Tinta Barroca grape varieties, mainly focused on the composition of monoterpenes, sesquiterpenes, and C_13_-norisoprenoids. Additionally, this work will also assess the usefulness of flow modulator-based GC × GC-TOFMS on the tracing signature of the grape varieties under study.

## 2. Results

### 2.1. Oenological Parameters of Grape Samples

Table 1 shows the oenological parameters of different red grape samples. For Trinc, CS, and Cast, the potential alcohol degree was almost equal for both years (only 0.1%vol), while a difference of 0.7 and 0.3 (%vol) was found in TB and Sy; Sy was the only variety presenting a higher value in 2021. All values of total acidity and pH are within normal values for these varieties.

### 2.2. Optimization of HS-SPME-GC × GC-TOFMS Methodology

The efficiency of HS-SPME extraction depends on several experimental parameters, such as the fiber coating, extraction time, temperature, sample amount, sample dilution, and amount of salt (NaCl). So, to increase the effectiveness of this technique, a detailed optimization of each step was carried out and the best conditions have been chosen according to the total number of monoterpenes, sesquiterpenes, and C_13_-norisorprenoids identified, because these compounds play a key role in the differentiation of the viniferous varieties.

The extraction efficiency of the sample preparation technique greatly depends on the value of the distribution constant of analytes partitioned between the sample and the fiber coating material; consequently, the selection of a suitable fiber coating is crucial. The DVB/CAR/PDMS fiber has been described in the literature as having a good affinity for the monoterpenes and C_13_-norisorprenoids with a lower and higher boiling point [36]. According to Rebière et al.’s 2010 study [37], these fibers ensure the extraction of compounds with an extensive range of molecular masses (40–275 m/z). Thus, the 50/30 μm DVB/CAR/PDMS fiber was automatically selected for the adsorption of the target compounds in this study.

**Figure 1:**
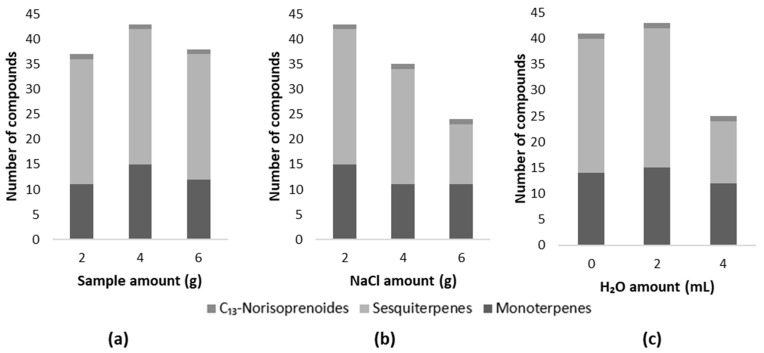
Influence of the different amounts of (**a**) sample, (**b**) salt (NaCl), and (**c**) H_2_O in the number of terpene compounds of each chemical family.

Initially, to optimize the sample quantity, 2, 4, and 6 g of red grapes were tested. Figure 1a shows the results obtained for each quantity in the study. Based on the findings, employing 2 g of the sample led to the identification of thirty-seven compounds (twenty-five sesquiterpenes, ten monoterpenes, and two C_13_-norisoprenoids). Increasing the sample amount to 4 g resulted in the detection of forty-three compounds (twenty-seven sesquiterpenes, fourteen monoterpenes, and two C_13_-norisoprenoids). Likewise, when utilizing 6 g of grapes, thirty-eight compounds were detected (twenty-five sesquiterpenes, eleven monoterpenes, and two C_13_-norisoprenoids). The decrease in the number of compounds when 6 g of grapes were used can be explained by the possible competition between the analytes of interest and the remaining volatile compounds in the fiber coating. Therefore, 4 g was selected as the sample amount in the following study. It is important to highlight that this study was performed in triplicate and the number of identified compounds was the same between repetitions.

The use of NaCl increases the ionic strength of the samples, and affects the decrease in the solubility of the compounds and their partition coefficient, improving the extraction of the analytes [38]. So, in this work 2, 4, and 6 g of salt were tested, starting with 2 g of NaCl, because this amount prevents the grape’s fermentation. In fact, the presence of fermentative compounds could decrease the sensitivity of the fiber by competing with the target analytes at the time of adsorption. Figure 1b shows that the increase in the amount of salt leads to a loss in the number of terpene compounds identified. This can be explained by the fact that the use of NaCl favors the release of polar compounds occupying the binding sites on the fiber to the detriment of terpenes, which are compounds possessing a more non-polar nature [39]. This decrease is also reported in the literature and indicates that the increase in the amount of salt can indeed lead to a loss of fiber selectivity [23].

According to Perestrelo et al. 2011 [4], when we are dealing with complex matrices such as grapes and wines, sample dilution can affect the partition between the headspace and the sample. Consequently, the volatile profile can be altered, as competition between the analytes that are presented in the headspace [4]. To investigate the dynamics of headspace competition, we conducted sample dilution by adding 2 and 4 mL of H_2_O. A sample without dilution was used for comparison. The results obtained are presented in Figure 1c, and it is possible to observe that, using 2 mL of H_2_O, it was possible to identify 45 compounds. Using 4 mL of H_2_O allowed for the identification of 25 compounds, while 41 compounds were identified in the undiluted sample. This result was relatively unexpected, since according to the literature [4], a more significant difference should have been observed between the undiluted sample and the sample with 2 mL of H_2_O, which allows us to infer that this dilution did not affect partitioning between the headspace and the sample. However, with a higher dilution of 4 mL, it was observed that the number of compounds found decreased, specially the sesquiterpenes. Nevertheless, the results show that the addition of 2 mL of H_2_O was the most promising procedure for the analysis of varietal volatile compounds.

**Figure 2:**
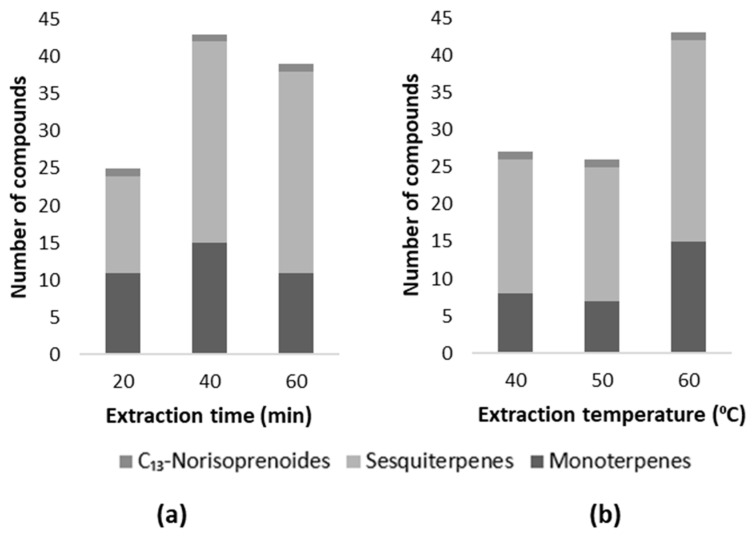
Influence of the (**a**) extraction time and (**b**) extraction temperature on the number of terpene compounds of each chemical family.

After optimizing the amount of the sample, salt, and H_2_O, the extraction time and temperature were optimized. In order to optimize the extraction time, the DVB/CAR/PDMS fiber was exposed in the headspace for 20, 40, and 60 min. The choice of extraction times was based on the information in the literature [4]. According to Figure 2a, it appears that 40 min of extraction is the optimal time, since it is possible to identify a greater number of compounds, and for this reason this was the time chosen for the implementation of the methodology. In addition, the extraction temperature was also optimized, since it affects the extraction efficiency of the volatile compounds. According to the literature [4], terpenes and C_13_-norisoprenoids show better results at higher extraction temperatures. To evaluate this condition, the samples were tested at 40, 50, and 60 °C, as according to Perestrelo et al. 2011 [4]. The results obtained are presented in Figure 2b. It can be observed that the best extraction temperature used was 60 °C, as this allowed for the identification of 43 varietal volatile compounds.

### 2.3. Application of HS-SPME-GC × GC-TOFMS Methodology for Analysis of Varietal Volatile Composition

After the optimization steps, the global volatile signature of the grapes from Trinc, CS, Sy, Cast, and TB grapes was established. The contour plots of Trinc, CS, Sy, Cast, and TB grapes are shown in Figures S1, S2, S3, S4 and S5, respectively.

Figure S6 shows a contour plot of a sample of grapes in the study, with the assignments of monoterpenes, C_13_-norisoprenoids, and sesquiterpenes regions. As expected, the compounds are eluted from the chromatographic system according to their affinity to the column; thus, the monoterpenes (region (a)) come out first, followed by the C_13_-norisoprenoids (region (b)), and finally the sesquiterpenes (region (c)), C_10_, C_13_, and C_15_, respectively.

In the grape varieties under analysis, it was possible to identify and quantify a total of fifty-two free varietal volatile compounds, with seventeen compounds identified as belonging to the monoterpene class, twenty-eight compounds assigned to the sesquiterpene family, and seven compounds attributed to the C_13_-norisoprenoid group (Table 2).

Compounds like p-cymene, d-limonene, p-cymenene, linalool, α-copaene, α-gurjunene, longifolene, β-caryophyllene, α-humulene, α-cadinene, β-cadinene, cis-calamenene, α-calacorene, cadalene, β-cyclocitral, geranylacetone, and β-ionone were found in all the varieties studied. However, certain compounds are exclusive to specific varieties. For instance, β-myrcene, hotrienol, and β-citronellol are exclusively found in the TB variety, while β-elemene, α-cedrene, and aromadendrene are unique to the Sy variety. Additionally, isocaryophyllene is solely present in the Trinc variety. Moreover, the compounds γ-elemene, γ-muurolene, and α-amorphene are present in all varieties except TB, while tetrahydrolinalool is present in all varieties under study, except Trinc.

As expected, an inter-annual variability is observed; therefore, there are compounds that are only present, for example, in 2021 or 2022. In the case of the Cast variety compounds, such as, γ-terpinene, α-terpinolene, γ-terpineol, α-terpineol, vitispirane, theaspirane B, and β-damascenone, they are only present in the year 2021 and were not found in 2022. However, compounds such as dihydromyrcenol, δ-elemene, aristolene, β-copaene, β-guaiene, zonanere, and the unknown compound m/z 105/161/189/204 are only present in the year 2022 and not in 2021. A similar trend is observed for the other varieties under study; for example, in TB, the compounds cis-myrtanol and γ-selinene were only found in 2021 and not in 2022, but α-ylangene and β-damascenone were only found in 2022 and not in 2021. In Sy grapes, compounds like γ-terpinene, α-terpinolene, γ-terpineol, and cis-myrtanol were only identified in the year 2021, and nerol and δ-selinene were identified in the year 2022 but not in 2021. As for the CS variety, compounds such as γ-terpinene, α-terpinolene, γ-terpineol, nerol, cis-myrtanol, γ-selinene, and β-copaene were only found in the year 2021, while valencene was only found in the year 2021. In the case of the Trinc variety, it also presents compounds that are only present in 2022 and not in 2021, such as δ-elemene, α-cubebene, α-ylangene, aristolene, and δ-selinene.

According to van Leeuwen et al. 2020 [40], volatile compounds are rarely assigned as variety markers. Indeed, the concentration of volatile compounds changes from variety to variety, but other factors such as the effect of the terroir, climatic conditions, and harvest dates also have to be considered due to their remarkable effect on the volatile composition of the grapes. According to Petronilho et al. 2021 [41], the same volatile compounds were found in different varieties, revealing that the individual aromatic potential of these varieties is related to infinite combinations and modulated by environmental characteristics. Other studies also corroborate the results obtained in this work, showing differences in the volatile varietal composition of grapes between years and between varieties [1,4,42,43].

**Figure 3:**
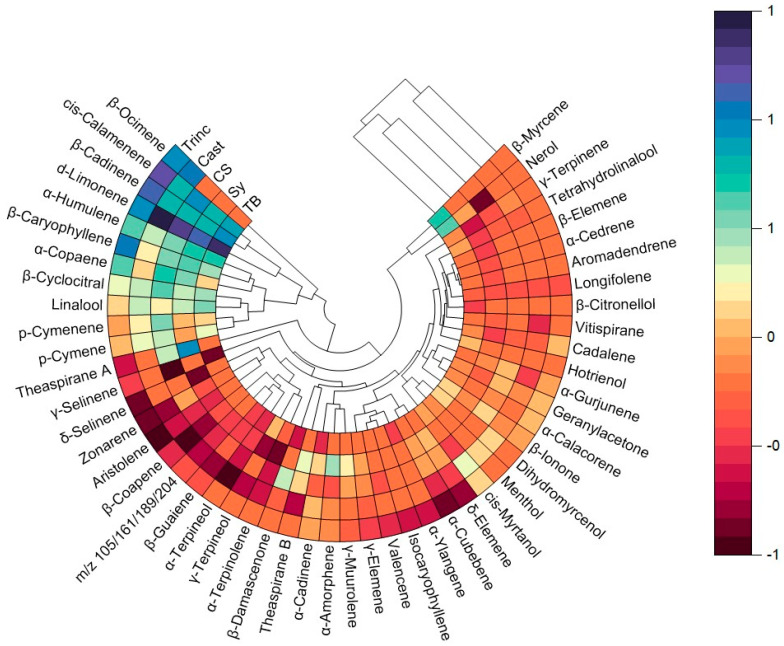
Polar heatmaps with dendrogram of free varietal volatile compounds identified in five varieties, Cast, Trinc, CS, Sy, and TB.

Figure 3 shows the polar heatmaps with a dendrogram for the varieties under study. These data evidenced differences between varieties and, apparently, there seems to be a clear hierarchical grouping between the Portuguese varieties Trinc, Cast, and TB, and between the CS and SY varieties. Another interesting finding is the similarity, in terms of volatile composition, between Tinc and Cast varieties, which are two of the most used and ancient varieties in the Alentejo region. As depicted in Figure 3, the compounds were grouped into five clusters; β-ocimene, the largest compound in all varieties, is included in cluster 1. Therefore, the compounds cis-calamenene, β-cadinene, and d-limonene are included in cluster 2, α-humulene, β-caryophyllene, α-copaene, β-cyclocitral, linalool, p-cymenene, and p-cymene belong to cluster 3, theaspirane A belongs to cluster 4, and the other identified compounds belong to cluster 5, which are present in trace levels.

For the monoterpene class, attending to the relative area, it is observed that Cast was the variety that presented a higher total relative area, 69.34 ± 4.32%, while Trinc presented the lowest total relative area, 20.27 ± 3.48%. For the sesquiterpene family, Trinc was the variety that presented a higher total relative area, 75.29 ± 6.94%, while Cast showed a smaller total relative area, 20.70 ± 3.24%. Particularly, for the C_13_-norisoprenoid family, the variety that showed the largest total relative area was Sy, with 23.63 ± 3.91%; Trinc and TB were the varieties that showed the smallest relative area, with 4.44 ± 0.59% and 4.76 ± 0.94%, respectively.

The results of the ANOVA and MANOVA presented in Table S1 assess the effects of the year, variety, and interaction between the year and variety, which vary across the families of compounds under study. In the case of monoterpenic compounds, the year, variety, and year × variety interaction effects are not statistically significant for the majority of the compounds, except for p-cymene, d-limonene, and linalool, which are statistically significant. As for the C_13_-norisoprenoids, the year did not significantly affect most of the compounds, with the exception of theaspirane A and theaspirane B. The variety effect was statistically significant for almost all C_13_-norisoprenoids, except for vitispirane. The year × variety interaction effect was significant for almost all C_13_-norisoprenoids except for vitispirane and β-damascenone. As for the sesquiterpenic compounds, regarding the effect of the year, in general, most compounds are statistically significant. As for the effect of variety, 50% of the sesquiterpenic compounds are not statistically significant, but the remaining 50% of these compounds are statistically significant. The interaction year × variety interaction for these compounds was not significant. The results of the ANOVA showed that the effect of the year was not statistically significant for the compounds that are present in both years.

### 2.4. Linear Discriminant Analysis (LDA)

To perform the LDA, statistically significant compounds (*p* < 0.05) were selected. The LDA is a method that relates quantitative independent variables (relative areas of compounds) with qualitative dependent variables (varieties under study) through discriminant functions to explain differences between varieties [44].

**Figure 4:**
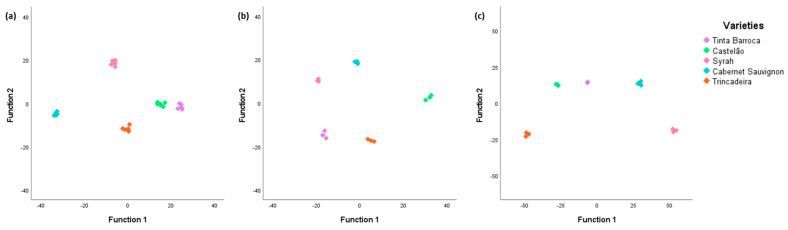
Linear discriminant analysis (represented by canonical discriminant functions) of grapes of five varieties for (**a**) both years, (**b**) from 2021, and (**c**) from 2022, based on significantly different relative areas.

For this analysis, we employed four discriminant functions, which were evaluated based on their significance and Wilks lambda factor. The Wilks lambda factor allows for the testing of the significance of the discriminant functions for a significance level of 0.05. The Wilks lambda range varies from 0 to 1 and the closer to zero the Wilks lambda value is, the more distinct the varieties are [45]. In the case of varieties (Figure 4a), it was found that all functions had significance levels <0.001, which suggests that the discriminant functions are significantly different. For the 2021 (Figure 4b) and 2022 (Figure 4c) varieties, the Wilks lambda values obtained showed significance levels <0.001 for functions 1, 2, and 3. Function 4 exhibited significance levels of 0.027 and 0.005 for the varieties in the years 2021 and 2022, respectively, indicating significant differences among the discriminant functions once more.

The eigenvalues indicate how different the varieties are according to the discriminant functions; that is, for the results of Figure 4a, function 1 explains 73.1%, function 2 explains 19.3%, function 3 explains 5.5%, and function 4 explains 2.1%. The Wilks lambda value is 0 for the first 2 functions and 0.002 and 0.068 for function 3 and 4, respectively. Thus, the first 3 functions have good discriminatory power. As for the results of the 2021 data set, function 1 explains 58.9%, function 2 explains 34.4%, function 3 explains 5.5%, and function 4 explains 1.2%. The Wilks lambda value is 0 for the first two functions, while for functions 3 and 4, it is 0.002 and 0.089, respectively, indicating that only three functions possess high discriminatory power. As for the results for the 2022 data set, function 1 explains 79.8%, function 2 explains 16.1%, function 3 explains 3.3%, and function 4 explains 0.9%. The Wilks lambda value is 0 for the first 2 functions and 0.001 and 0.043 for function 3 and 4, respectively. Therefore, all functions have good discriminatory power. The application of the LDA demonstrated statistical differences among the five varieties studied, both in 2021 and 2022.

## 3. Materials and Methods

### 3.1. Samples

Five different V. vinifera red grape varieties in two different years were studied. Grapes of Trincadeira (Trinc), Cabernet Sauvignon (CS), Syrah (Sy), Castelão (Cast) and Tinta Barroca (TB) varieties were harvested in 2021 and 2022 from the experimental vineyard of Évora University.

Oenological parameters, such as potential alcohol degree, total acidity, and pH were measured according to the OIV (International Organization of Vine and Wine) [46].

### 3.2. HS-SPME Sampling Conditions

To increase the efficiency of the SPME technique, a detailed optimization of all the steps of the sample preparation technique was carried out. Different conditions—the amount of the sample (2, 4, and 6 g of grapes), salt amounts (2, 4, and 6 g of NaCl), H_2_O volumes (0, 2, and 4 mL), time of extraction (20, 40 and 60 min), and extraction temperature (40, 50, and 60 °C) were considered. All measurements were performed with three replicates.

The optimization of the different stages of the SPME allowed for the implementation of the following methodology: A carboxen/divinylbenzene/polydimethylsiloxane fiber (CAR/DVB/PDMS), 1 cm, 50/30 μm film thickness, supplied from Supelco, (Bellefonte, PA, USA) was used for HS-SPME extractions. Fiber blanks were run periodically, that is, a blank was carried out before the injection of the first sample of grapes and the remaining blanks were carried out every 3 injections, to ensure the absence of contaminants and/or carryover. HS-SPME extraction was performed according to following procedure; 4 g of each grape sample was crushed with the Ultra Turrax T25 basic homogenizer, (IKA Labortechnik, Germany); then, 2 g sodium chloride (NaCl) and finally 2 mL of ultra-pure water were introduced in a 20.0 mL SPME vial and sealed with a Teflon-lined rubber septum/magnetic screw cap; the vial was equilibrated for 5 min at 60 °C and then extracted for 40 min at the same temperature. The thermal desorption of the analytes was carried out by exposing the fiber in the GC injection port at 260 °C for 3 min in splitless mode. All measurements were performed in triplicate.

### 3.3. GC × GC-TOFMS Analysis

The analyses were performed on a GC × GC-TOFMS system consisting of an Agilent 8890GC System (Shanghai, China) with a BenchTOF-Select detector (Markes International, Bridgend, UK). An automatic sampler injector was used (CTC Analysis AG autosampler PAL-System, SepSolve Analytical, Zwingen, Switzerland) and the data were acquired and analyzed with ChromSpace of Markes International. Chromatographic separation was achieved with the INSIGHT™ flow modulator (SepSolve Analytical), equipped with a loop with 50 µL, a BPX5 column (20 m length × 0.18 mm i.d. and 0.18 μm film thickness, SGE GC column, Trajan, Australia) as the first dimension (1D), and a BPX50 column (5 m length × 0.25 mm i.d. and 0.1 μm film thickness, SepSolve Analytical, Australia) as the second dimension (2D). The modulation period (PM) used was 5s. The oven temperature program began at 40 °C hold for 3 min; then, the temperature was raised at 3 °C min^−1^ up to 150 °C, then 4 °C min^−1^ up to 260 °C, and held for 10 min. Helium was used as carrier gas with a flow of 0.5 mL min^−1^ in the first column and 20 mL min^−1^ in the second column. The MS transfer line and source temperatures were set at 270 °C. Spectra were matched using the NIST MS Search Program Version 2020. To determine the retention times and characteristic mass fragments, electron ionization (EI) at 70 eV mass spectra of the analytes were recorded at full scan, from 30 to 400 m/z, and a data acquisition frequency of 50 Hz. The linear retention index values were calculated through an analysis of the commercial hydrocarbon mixture (C_8_–C_20_) [Supelco, Bellefonte, PA, USA], using the same chromatographic conditions. A mixture of terpenes called MegaMix #1 (Restek) were injected to help identify terpenes. The volatile compounds were first identified by matching the mass spectra with the spectra of the reference compounds in the NIST mass spectral library, also taking into consideration the structure and molecular weight, and by comparing the calculated LRIs with those described in the literature. The relative amount of each compound was calculated as the percent ratio of the respective peak area relative to the total peak area and expressed as percentage (%).

### 3.4. Statistical Analysis

IBM SPSS Statistics 27 was used to perform all statistical analyses. A multivariate analysis of variance (MANOVA) was used to assess whether the variables “variety”, “year”, or the interaction “variety × year” were statistically significant. A one-way analysis of variance (ANOVA) was also used to test the differences between the years under study. Differences between groups were evaluated at a probability level of 0.05, 0.01, and 0.001, using the Fisher test. Then, a linear discriminant analysis (LDA) was performed on the data expressing the relative areas attributed to the varietal volatile compounds (independent variables) according to the variety (dependent variable), using only significantly different variables. For each discriminated function, the statistical significance was evaluated based on the Wilks lambda factor.

OriginPro 2023b SR1 (OriginLab, Northampton, MA, USA) was used to perform polar heatmaps.

## 4. Conclusions

HS-SPME combined with GC × GC-TOFMS provides a suitable and sustainable approach to establish the volatile signature of grapes of different varieties. The SPME technique was optimized, and the only conditions that were considered sufficient to obtain a good extraction were a duration of 40 min and a temperature of 60 °C. The optimization of the modulation conditions was also crucial to guarantee the success of the analysis.

Overall, seventeen monoterpenes, twenty-eight sesquiterpenes, and seven C_13_-norisoprenoids were found. Compounds such as β-myrcene, hotrienol, and β-citronellol are exclusive to the TB variety, while β-elemene, α-cedrene, and aromadendrene are specific to the Sy variety. Isocaryophyllene, on the other hand, is solely found in the Trinc variety. In 2021, TB exhibited a higher relative area for the total sum of free varietal volatile compounds compared to the other varieties under study. In the year 2022, it was the Trinc variety that obtained a higher total relative area.

The application of the LDA demonstrated statistically significant differences among the five varieties under study, both in 2021 and 2022.

The work described herein marks the initial phase of a comprehensive study on varietal volatiles within selected grape varieties cultivated in Portugal. The subsequent phase will naturally involve analyzing bound varietal volatile compounds, as they constitute a reservoir of compounds that may be liberated into wine following hydrolysis during wine production processes.

## Figures and Tables

**Table 1:** Chemical characterization of different red grape samples.

	Year	Potential Alcohol Degree (% vol)	pH	Total Acidity (g L^−1^) ^1^
Trinc	2021	12.9	3.17	5.51
	2022	13.0	4.70	3.60
CS	2021	13.1	3.11	7.82
	2022	13.2	3.40	6.72
TB	2021	13.6	3.66	4.74
	2022	14.3	3.59	4.27
Sy	2021	13.6	3.43	4.89
	2022	13.3	3.40	5.84
Cast	2021	12.9	3.31	5.35
	2022	13.0	3.66	5.44

^1^ Tartaric acid.

**Table 2:** Free varietal volatile compounds found in samples of Trinc, CS, TB, Sy, and Cast grapes, mean and standard deviation of relative areas.

Compounds	^1^Dt_r_ (min) ^a^	^2^Dt_r_ (min) ^b^	LRI _calc_ ^c^	LRI _lit_ ^d^	Varieties 2021					Varieties 2022				
					Trinc	CS	TB	Sy	Cast	Trinc	CS	TB	Sy	Cast
Monoterpenic Compounds														
β-Myrcene ^e^	19.32	3.00	986	992	nd	nd	6.83 ± 3.96	nd	nd	nd	nd	6.33 ± 4.09	nd	nd
p-Cymene ^e^	21.31	3.34	1024	1031	2.31 ± 0.42	3.69 ± 0.24	2.16 ± 0.28	1.60 ± 0.33	4.19 ± 0.15	1.06 ± 0.43	2.74 ± 0.45	3.81 ± 1.51	1.27 ± 0.12	1.76 ± 0.64
d-Limonene ^e^	21.50	3.12	1027	1035	15.01 ± 6.03	19.55 ± 14.55	25.25 ± 2.51	15.62 ± 3.80	45.13 ± 1.52	4.45 ± 0.89	15.71 ± 6.89	17.59 ± 17.36	10.91 ± 5.08	10.94 ± 0.29
Ocimene ^e^	21.60	3.17	1037	1037	9.78 ± 4.52	nd	nd	nd	5.63 ± 0.35	10.49 ± 1.53	nd	nd	nd	18.47 ± 2.54
γ-Terpinene ^e^	23.03	3.19	1054	1065	nd	1.52 ± 0.35	1.03 ± 0.28	0.89 ± 0.60	2.64 ± 0.07	nd	nd	1.30 ± 0.30	nd	nd
Dihydromyrcenol	23.78	3.24	1066	1080	nd	nd	1.43 ± 0.22	nd	nd	nd	nd	2.67 ± 0.87	nd	3.85 ± 0.21
α-Terpinolene ^e^	24.39	3.23	1082	1092	nd	0.91 ± 0.26	0.80 ± 0.46	0.64 ± 0.07	0.93 ± 0.57	nd	nd	0.95 ± 0.52	nd	nd
p-Cymenene	24.87	3.53	1076	1101	1.88 ± 0.38	7.62 ± 5.74	1.40 ± 0.07	1.33 ± 0.13	2.11 ± 0.07	1.05 ± 0.29	1.30 ± 0.68	2.68 ± 0.47	2.03 ± 0.18	2.31 ± 0.81
Tetrahydrolinalool	25.09	3.06	1091	1106	nd	0.65 ± 0.20	0.79 ± 0.19	0.59 ± 0.07	0.67 ± 0.14		1.06 ± 0.16	1.97 ± 1.09	0.66 ± 0.21	1.03 ± 0.21
Linalool ^e^	25.17	3.33	1086	1107	2.83 ± 0.32	1.86 ± 0.51	3.28 ± 0.43	2.72 ± 0.30	3.02 ± 0.36	1.24 ± 0.23	2.99 ± 0.26	6.07 ± 1.91	3.78 ± 0.60	3.39 ± 0.32
Hotrienol	25.42	3.41	1107	1112	nd	nd	0.40 ± 0.17	nd	nd	nd	nd	3.11 ± 3.56	nd	nd
γ-Terpineol	26.58	3.22	1170	1135	nd	nd	1.25 ± 0.57	0.66 ± 0.10	0.83 ± 0.08	nd	nd	2.11 ± 0.94	nd	nd
Menthol ^e^	29.37	3.32	1169	1191	nd	nd	0.72 ± 0.47	nd	1.20 ± 0.26	nd	nd	1.71 ± 1.16	nd	3.09 ± 0.37
α-Terpineol ^e^	30.25	3.56	1182	1209	nd	0.90 ± 0.14	0.28 ± 0.08	0.65 ± 0.19	0.50 ± 0.16	nd	nd	0.78 ± 0.23	0.63 ± 0.03	nd
β-Citronellol ^e^	31.50	3.44	1214	1236	nd	nd	0.54 ± 0.20	nd	nd	nd	nd	0.82 ± 0.36	nd	nd
Nerol ^e^	32.66	3.54	1218	1261	nd	0.64 ± 0.09	4.62 ± 0.36	nd	nd	nd	nd	5.78 ± 3.20	2.92 ± 0.37	nd
cis-Myrtanol	34.42	3.43	1261	1299	2.05 ± 0.64	1.42 ± 0.50	2.90 ± 1.13	2.17 ± 0.51	2.48 ± 0.58	1.97 ± 0.11	nd	nd	2.90 ± 1.13	3.40 ± 0.47
Total					33.86 ± 12.29	38.76 ± 22.56	53.67 ± 11.39	26.88 ± 6.12	69.34 ± 4.32	20.27 ± 3.48	23.80 ± 8.44	57.69 ± 37.57	22.20 ± 6.59	48.23 ± 5.58
Sesquiterpenic compounds														
δ-Elemene	36.42	3.05	1343	1343	nd	1.13 ± 0.18	nd	1.37 ± 0.16	nd	0.72 ± 0.11	1.87 ± 0.44	nd	1.99 ± 0.22	1.38 ± 0.66
Compounds	^1^Dt_r_ (min) ^a^	^2^Dt_r_ (min) ^b^	LRI _calc_ ^c^	LRI _lit_ ^d^	Varieties 2021					Varieties 2022				
					Trinc	CS	TB	Sy	Cast	Trinc	CS	TB	Sy	Cast
α-Cubebene	36.94	3.02	1358	1354	nd	0.99 ± 0.13	nd	1.38 ± 0.15	0.40 ± 0.02	0.55 ± 0.03	1.85 ± 0.23	nd	1.47 ± 0.60	0.82 ± 0.11
α-Ylangene	38.00	3.10	1376	1378	nd	nd	nd	0.72 ± 0.11	nd	0.98 ± 0.37	nd	1.32 ± 0.75	1.52 ± 0.33	nd
α-Copaene	38.33	3.10	1380	1385	4.24 ± 0.35	4.96 ± 0.83	2.19 ± 1.10	3.74 ± 2.11	1.53 ± 0.06	5.80 ± 0.31	7.60 ± 0.65	1.88 ± 0.33	4.79 ± 0.56	2.83 ± 0.33
β-Elemene	38.94	3.17	1388	1399	nd	nd	nd	0.50 ± 0.12	nd	nd	nd	nd	0.90 ± 0.40	nd
Isocaryophyllene	39.67	3.26	1403	1417	0.61 ± 0.02	nd	nd	nd	nd	0.40 ± 0.19	nd	nd	nd	nd
α-Gurjunene	39.75	3.18	1406	1419	1.37 ± 0.14	1.44 ± 0.20	0.95 ± 0.36	1.19 ± 0.13	0.49 ± 0.12	1.70 ± 0.14	1.95 ± 0.39	1.10 ± 0.36	1.25 ± 0.21	0.77 ± 0.04
Longifolene	40.08	3.25	1409	1427	0.99 ± 0.75	0.61 ± 0.09	0.64 ± 0.31	0.72 ± 0.04	nd	0.57 ± 0.13	0.93 ± 0.21	1.18 ± 0.93	0.86 ± 0.21	1.09 ± 0.40
α-Cedrene ^e^	40.25	3.22	1414	1431	nd	nd	nd	0.66 ± 0.07	nd	nd	nd	nd	0.91 ± 0.10	nd
β-Caryophyllene ^e^	40.37	3.23	1424	1434	14.00 ± 1.85	4.47 ± 1.46	4.75 ± 3.44	5.86 ± 0.93	1.72 ± 0.41	9.62 ± 0.48	4.90 ± 0.80	3.08 ± 2.99	5.51 ± 0.48	2.87 ± 0.88
γ-Elemene	40.58	3.18	1432	1439	0.60 ± 0.03	0.93 ± 0.11	nd	1.10 ± 0.13	0.63 ± 0.06	0.67 ± 0.08	1.67 ± 0.07	nd	1.88 ± 0.47	1.17 ± 0.22
Aromadendrene	40.78	3.17	1447	1444	nd	nd	nd	0.71 ± 0.15	nd	nd	nd	nd	0.97 ± 0.45	nd
Aristolene	41.17	3.21	1429	1454	nd	0.45 ± 0.14	nd	0.37 ± 0.02	nd	0.48 ± 0.03	0.66 ± 0.04	nd	0.78 ± 0.07	0.90 ± 0.20
β-Coapene	41.58	3.20	1434	1464	0.54 ± 0.05	0.67 ± 0.49	nd	0.52 ± 0.01	nd	0.53 ± 0.06	nd	nd	1.16 ± 0.16	0.53 ± 0.05
α-Humulene ^e^	41.93	3.24	1456	1472	3.58 ± 0.68	4.07 ± 1.08	6.69 ± 3.08	6.03 ± 0.68	2.06 ± 0.36	6.31 ± 0.09	4.85 ± 0.91	6.01 ± 1.71	6.04 ± 0.77	4.22 ± 0.45
β-Guaiene	42.15	3.21	1482	1478	0.59 ± 0.11	0.68 ± 0.25	0.72 ± 0.19	0.57 ± 0.24	nd	0.96 ± 0.13	1.19 ± 0.02	0.67 ± 0.38	0.83 ± 0.38	0.65 ± 0.13
γ-Selinene	42.41	3.23	1486	1484	0.55 ± 0.05	0.47 ± 0.05	0.62 ± 0.25	nd	nd	0.73 ± 0.22	nd	nd	nd	nd
Valencene ^e^	42.50	3.21	1487	1487	0.48 ± 0.08	nd	nd	nd	nd	0.65 ± 0.54	1.56 ± 1.31	nd	nd	nd
γ-Muurolene	42.62	3.21	1478	1489	0.64 ± 0.24	1.38 ± 0.63	nd	1.50 ± 0.45	0.77 ± 0.28	1.13 ± 0.27	1.34 ± 0.52	nd	3.50 ± 0.57	1.35 ± 0.18
α-Amorphene	42.71	3.15	1479	1492	0.89 ± 0.02	0.59 ± 0.16	nd	6.26 ± 8.17	0.79 ± 0.13	1.65 ± 0.39	0.90 ± 0.14	nd	2.01 ± 0.61	1.76 ± 0.30
δ-Selinene	43.00	3.22	1506	1499	nd	nd	nd	nd	nd	0.55 ± 0.25	nd	nd	0.64 ± 0.05	nd
Zonarene	43.33	3.20	1530	1508	nd	0.64 ± 0.12	nd	0.61 ± 0.09	nd	0.63 ± 0.18	2.17 ± 0.12	nd	0.83 ± 0.04	0.74 ± 0.12
α-Cadinene	43.35	3.20	1522	1508	0.87 ± 0.09	0.59 ± 0.04	0.44 ± 0.11	1.16 ± 0.32	0.77 ± 0.31	2.25 ± 0.91	1.10 ± 0.18	0.65 ± 0.08	3.18 ± 1.49	2.86 ± 0.50
β-Cadinene	44.15	3.23	1522	1530	10.56 ± 2.28	6.48 ± 1.94	12.23 ± 1.76	6.21 ± 0.33	4.93 ± 0.67	15.87 ± 0.25	8.21 ± 0.86	7.27 ± 0.50	9.16 ± 1.24	10.18 ± 0.57
cis-Calamenene	44.40	3.33	1531	1537	13.18 ± 2.09	9.19 ± 3.34	9.90 ± 1.57	6.30 ± 0.56	4.48 ± 0.55	17.87 ± 1.24	11.56 ± 1.51	6.24 ± 0.53	9.28 ± 0.58	9.49 ± 0.59
m/z 105/161/189/204	44.83	3.48	---	1547	0.61 ± 0.12	0.53 ± 0.23	nd	0.49 ± 0.10	nd	0.93 ± 0.10	0.65 ± 0.07	nd	0.81 ± 0.03	0.78 ± 0.07
α-Calacorene	45.17	3.49	1536	1557	1.51 ± 0.04	1.02 ± 0.33	1.26 ± 0.11	1.02 ± 0.22	0.93 ± 0.04	2.12 ± 0.23	1.61 ± 0.25	1.15 ± 0.12	1.15 ± 0.08	1.24 ± 0.17
Cadalene	49.65	3.67	1662	1700	1.74 ± 0.32	0.85 ± 0.39	1.18 ± 0.30	0.48 ± 0.12	nd	1.64 ± 0.20	0.93 ± 0.06	1.16 ± 0.45	0.81 ± 0.06	1.08 ± 0.12
Total					57.53 ± 9.31	42.16 ± 12.20	41.57 ± 12.58	49.49 ± 15.41	20.70 ± 3.24	75.29 ± 6.94	57.50 ± 8.79	31.71 ± 9.15	62.22 ± 10.17	46.72 ± 6.09
C_13_-Norisoprenoids														
β-Cyclocitral	31.46	3.89	1198	1236	3.94 ± 0.89	5.80 ± 0.63	2.56 ± 0.46	3.78 ± 0.21	3.80 ± 0.58	1.85 ± 0.26	5.06 ± 0.82	5.35 ± 1.45	2.60 ± 0.80	2.53 ± 0.61
Vitispirane	34.12	3.31	1286	1292	nd	1.14 ± 0.12	nd	1.18 ± 0.51	1.13 ± 0.39	-	0.92 ± 0.27	nd	0.65 ± 0.10	nd
Theaspirane A	35.00	3.36	1284	1311	0.47 ± 0.10	3.17 ± 0.41	nd	11.58 ± 1.94	nd	0.44 ± 0.11	3.44 ± 0.21	nd	7.37 ± 0.80	nd
Theaspirane B	35.75	3.38	1299	1328	nd	1.90 ± 0.47	nd	3.99 ± 0.66	0.78 ± 0.48	-	2.00 ± 0.11	nd	1.86 ± 0.31	nd
Compounds	^1^Dt_r_ (min) ^a^	^2^Dt_r_ (min) ^b^	LRI _calc_ ^c^	LRI _lit_ ^d^	Varieties 2021					Varieties 2022				
					Trinc	CS	Trinc	CS	Trinc	CS	Trinc	CS	Trinc	CS
β-Damascenone	38.60	3.80	1368	1392	nd	3.42 ± 0.30	nd	1.14 ± 0.22	1.74 ± 0.50	-	3.26 ± 0.72	1.02 ± 0.12	0.98 ± 0.22	nd
Geranylacetone	41.33	3.45	1431	1458	2.56 ± 0.26	1.52 ± 0.24	0.76 ± 0.20	0.75 ± 0.14	nd	1.04 ± 0.06	1.96 ± 0.29	1.64 ± 0.81	1.00 ± 0.04	1.27 ± 0.18
β-Ionone	42.73	3.63	1491	1492	1.63 ± 0.33	2.13 ± 0.28	1.44 ± 0.28	1.20 ± 0.23	1.43 ± 0.22	1.11 ± 0.16	2.07 ± 0.27	2.58 ± 1.08	1.12 ± 0.14	1.25 ± 0.26
Total					8.61 ± 1.58	19.08 ± 2.46	4.76 ± 0.94	23.63 ± 3.91	9.96 ± 2.28	4.44 ± 0.59	18.70 ± 2.70	10.59 ± 3.46	15.58 ± 2.39	5.05 ± 1.05

^a 1^Dt_r_ (min): first dimension retention time; ^b 2^Dt_r_ (min): second dimension retention time; ^c^ LRI_calc_: The linear retention index values were calculated through analysis of the commercial hydrocarbon mixture (C_8_–C_20_); ^d^ LRI_lit_: The linear retention index values from the literature for a 5% phenyl polysilphenylene-siloxane column; ^e^ Compounds confirmed by MegaMix #1 (Restek, Bellefont, PA). nd: not detected.

## Data Availability

The data presented in this study are available on request from the corresponding author.

## Supplementary Materials

The following supporting information can be downloaded at: https://www.mdpi.com/article/10.3390/molecules29091989/s1, Figure S1: Example of a contour plot obtained through GC × GC-TOFMS analysis of a sample of Trincadeira grapes; Figure S2: Example of a contour plot obtained through GC × GC-TOFMS analysis of a sample of Cabernet Sauvignon grapes; Figure S3: Example of a contour plot obtained through GC × GC-TOFMS analysis of a sample of Tinta Barroca grapes; Figure S4: Example of a contour plot obtained through GC × GC-TOFMS analysis of a sample of Syrah grapes; Figure S5: Example of a contour plot obtained through GC × GC-TOFMS analysis of a sample of Castelão grapes; Figure S6: Example of a contour plot obtained through GC × GC-TOFMS analysis of a sample of CS grapes, region of (a) monoterpenes, (b) C_13_-norisoprenoids, and (c) sesquiterpenes; Table S1: The results of ANOVA and MANOVA for the free varietal compounds.
