# Supplementary material for: Comprehensive Two-Dimensional Gas Chromatography with a TOF MS Detector—An Effective Tool to Trace the Signature of Grape Varieties
Source: Molecules. 2024 Apr 26;29(9):1989. doi: 10.3390/molecules29091989 (PMC11085376; doi:10.3390/molecules29091989)
Supplement: Supplementary file 1 [file molecules-29-01989-s001.zip › molecules-2953262-supplementary.pdf]

Comprehensive two-dimensional gas chromatography with a TOF MS detector – An effective tool to trace the signature of grape varieties

Daniela Fonseca [1], Nuno Martins [2], Raquel Garcia [2,3] and Maria João Cabrita [2,3,*]

[1] Mediterranean Institute for Agriculture, Environment and Development & Institute of Research and Advanced Training, University of Évora, Pólo da Mitra, Ap. 94, 7006-554 Évora, Portugal; daniela.fonseca@uevora.pt
[2] Mediterranean Institute for Agriculture, Environment and Development & Global Change and Sustainability Institute, University of Évora, Pólo da Mitra, Ap. 94, 7006-554 Évora, Portugal.; nmartins@uevora.pt
[3] Department of Crop Science, School of Science and Technology, University of Évora, Pólo da Mitra, Ap. 94, 7006-554 Évora, Portugal; raquelg@uevora.pt; mjbc@uevora.pt
* Correspondence: mjbc@uevora.pt; Tel.: +351-266-760-869

Supplementary Files

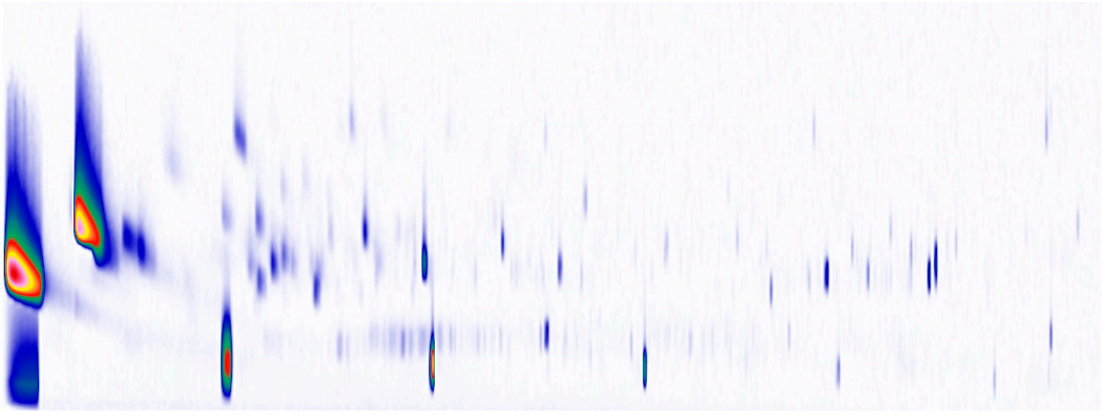

Figure S1. Example of a contour plot obtained through GC×GC-TOFMS analysis of a sample of grapes of Trincadeira.

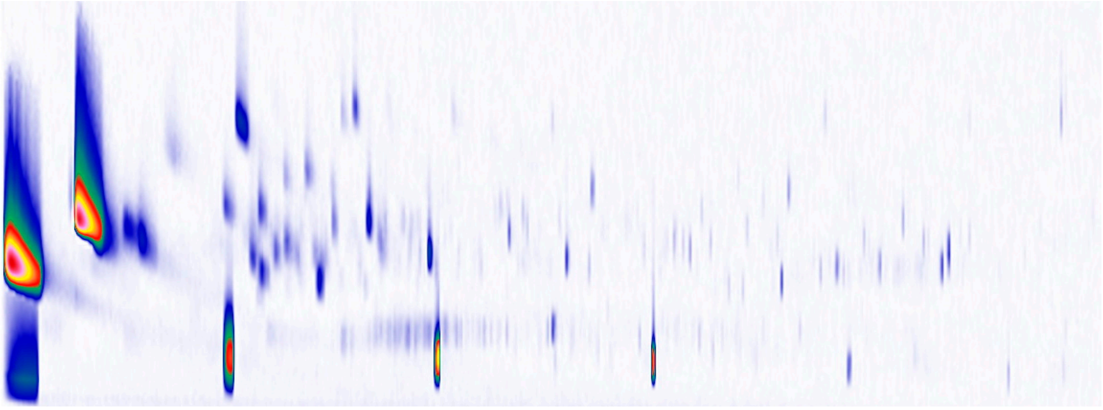

Figure S2. Example of a contour plot obtained through GC×GC-TOFMS analysis of a sample of grapes of Cabernet Sauvignon.

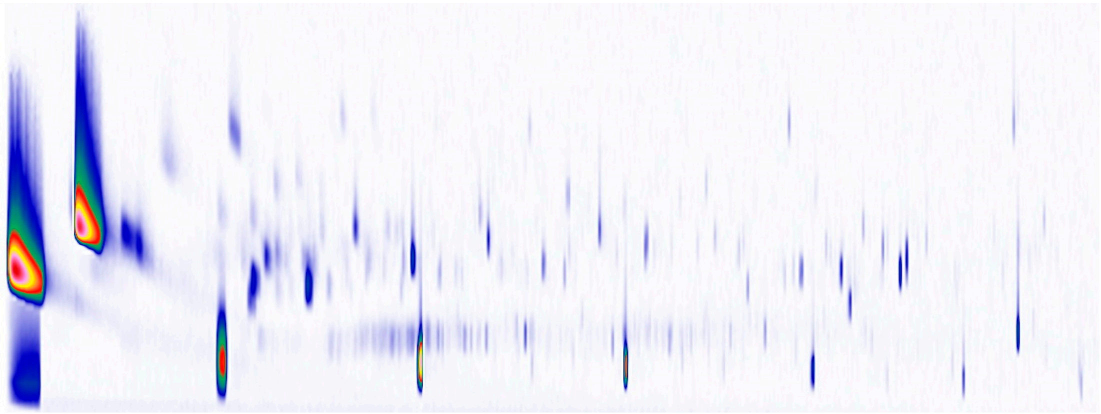

Figure S3. Example of a contour plot obtained through GC×GC-TOFMS analysis of a sample of grapes of Tinta Barroca.

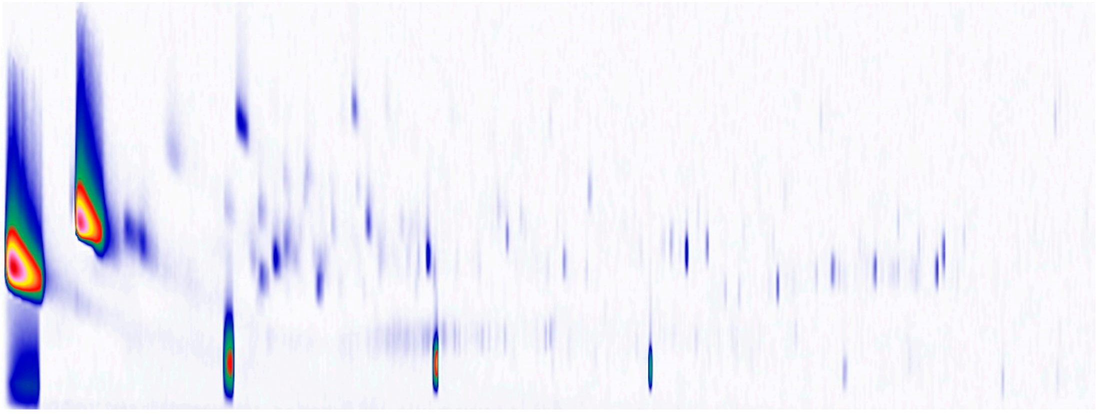

Figure S4. Example of a contour plot obtained through GC×GC-TOFMS analysis of a sample of grapes of Syrah.

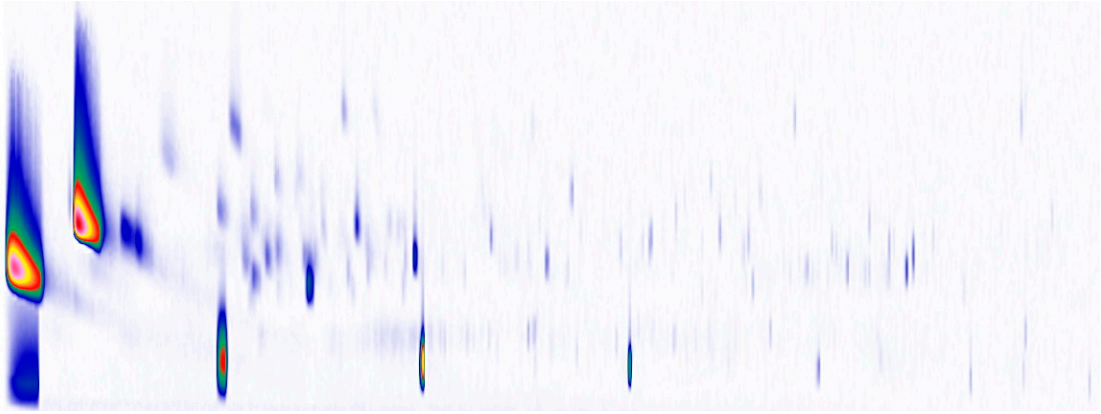

Figure S5. Example of a contour plot obtained through GC×GC-TOFMS analysis of a sample of grapes of Castelão.

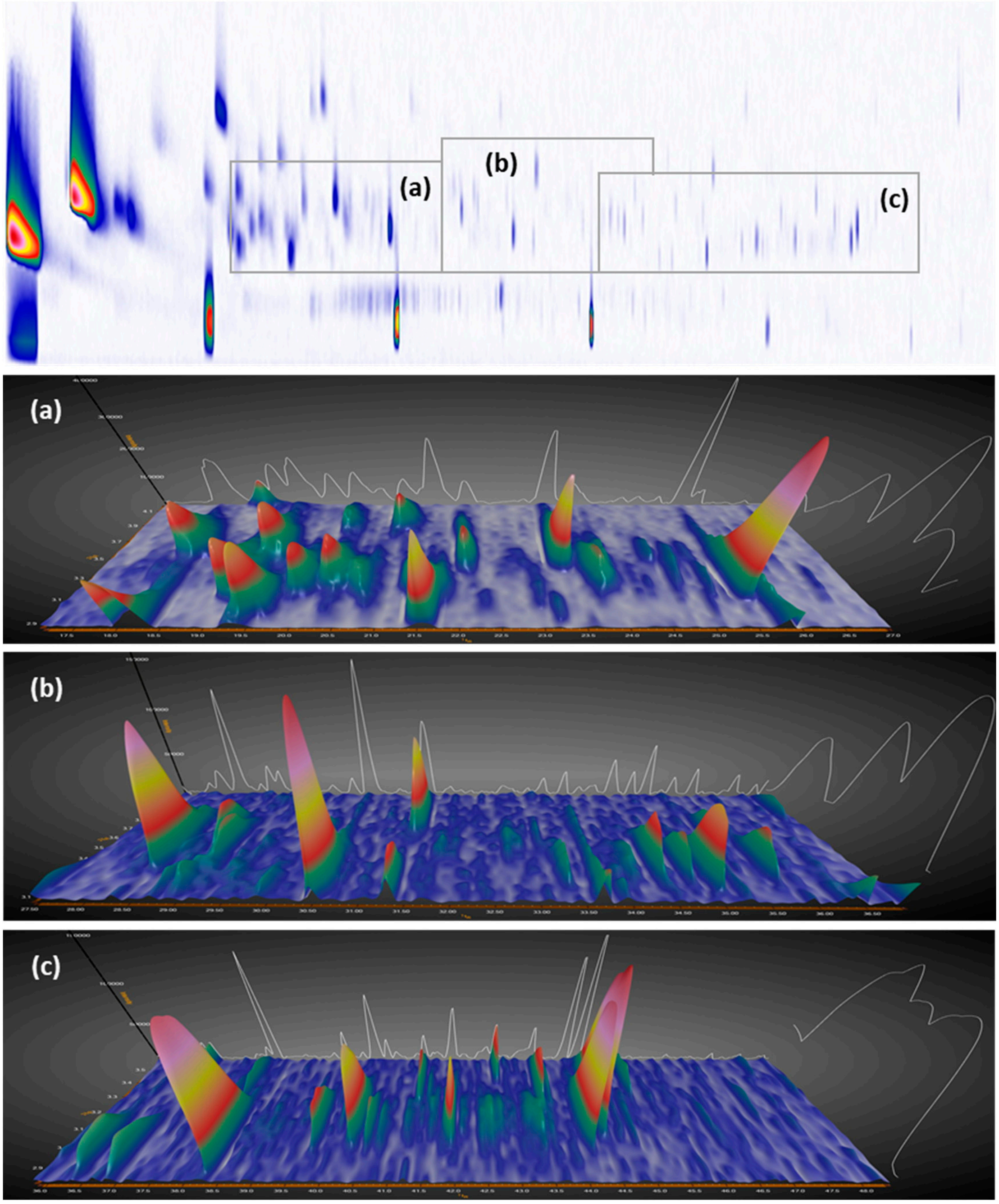

Figure S6. Example of a contour plot obtained through GC×GC-TOFMS analysis of a sample of grapes of CS, region of (a) monoterpenes, (b) C_{13}-norisoprenoids and (c) sesquiterpenes.

Table S1. The results of ANOVA and MANOVA for the free varietal compounds.

Compounds	ANOVA and MANOVA		
	Year	Variety	Year × Variety
**β-Myrcene**	NS	NS	NS
**p-Cymene**	**	***	***
**d-Limonene**	***	**	*
**Ocimene**	**	NS	**
**γ-Terpinene**	NS	NS	NS
**Dihydromyrcenol**	NS	NS	NS
**α-Terpinolene**	NS	NS	NS
**p-Cymenene**	NS	NS	*
**Tetrahydrolinalool**	**	*	NS
**Linalool**	**	***	***
**Hotrienol**	NS	NS	NS
**γ-Terpineol**	NS	NS	NS
**Menthol**	**	*	NS
**α-Terpineol**	*	NS	*
**β-Citronellol**	NS	NS	NS
**Nerol** [e]	NS	NS	NS
**cis-Myrtanol**	NS	*	NS
**δ-Elemene**	**	NS	NS
**α-Cubebene**	**	***	NS
**α-Ylangene**	*	NS	NS
**α-Copaene**	***	***	NS
**β-Elemene**	NS	NS	NS
**Isocaryophyllene**	NS	NS	NS
**α-Gurjunene**	**	***	NS
**Longifolene**	NS	NS	NS
**α-Cedrene**	*	NS	NS
**β-Caryophyllene**	NS	***	NS
**γ-Elemene**	***	***	*
**Aromadendrene**	NS	NS	NS
**Aristolene**	***	NS	NS
**β-Coapene**	***	***	***
**α-Humulene**	*	**	NS
**β-Guaiene**	*	NS	NS
**γ-Selinene**	NS	NS	NS
**Valencene**	NS	NS	NS
**γ-Muurolene**	***	***	**
**α-Amorphene**	NS	NS	NS
**δ-Selinene**	NS	NS	NS
**Zonarene**	***	***	***
**α-Cadinene**	***	***	*

Table S1. Cont.

Compounds	ANOVA and MANOVA		
	Year	Varieties	Year × Varieties
**β-Cadinene**	***	***	***
**cis-Calamenene**	***	***	***
**m/z 105/161/189/204**	**	NS	NS
**α-Calacorene**	***	***	*
**Cadalene**	NS	***	NS
**β-Cyclocitral**	NS	***	***
**Vitispirane**	NS	NS	NS
**Theaspirane A**	**	***	**
**Theaspirane B**	**	**	**
**β-Damascenone**	NS	***	NS
**Geranylacetone**	NS	***	***
**β-Ionone**	NS	**	*

Year×Variety: Interaction between year and varieties. Statistically significant at *$p \leq 0.05$, **$p \leq 0.01$ and ***$p \leq 0.001$, respectively. NS: Not significant.
